# In Vivo Nitrosative Stress‐Induced Expression of a Photolyase Promotes *Vibrio cholerae* Environmental Blue Light Resistance

**DOI:** 10.1111/mmi.15340

**Published:** 2025-01-15

**Authors:** Arkaprabha Banerjee, Hyuntae Byun, Andrew J. Hrycko, Qinqin Pu, Mary R. Brockett, Nathaniel C. Esteves, Jennifer R. Miller, Qiushi Li, Amy T. Ma, Jun Zhu

**Affiliations:** ^1^ Department of Microbiology, Perelman School of Medicine University of Pennsylvania Philadelphia Pennsylvania USA

**Keywords:** anti‐sigma factor, blue light, colonization, nitric oxide, pathogenesis, photolyase, RpoE, *Vibrio cholerae*

## Abstract

Bacterial pathogens possess a remarkable capacity to sense and adapt to ever‐changing environments. For example, *Vibrio cholerae* , the causative agent of the severe diarrheal disease cholera, thrives in aquatic ecosystems and human hosts through dynamic survival strategies. In this study, we investigated the role of three photolyases, enzymes that repair DNA damage caused by exposure to UV radiation and blue light, in the environmental survival of *V. cholerae* . Among these, we identified *cry1* as critical for resistance to blue light, as mutations in this gene, but not in the other photolyase genes, rendered *V. cholerae* susceptible to such stress. Expression of *cry1* was induced by blue light and regulated by RpoE and the anti‐sigma factor ChrR. We further showed that nitric oxide (NO), a host‐derived stressor encountered during infection, also activated *cry1* expression. We found that one of the two cysteine residues in ChrR was important for sensing reactive nitrogen species (RNS), thereby modulating *cry1* expression. While Cry1 was not required for *V. cholerae* colonization in animal models, pre‐induction of Cry1 by RNS in vivo or in vitro enhanced *V. cholerae* resistance to blue light. These findings suggest that host‐derived NO encountered during infection primes *V. cholerae* for survival in blue‐light‐rich aquatic environments, supporting its transition between host and environmental niches.

## Graphical abstract

This study demonstrates how *Vibrio cholerae* activates the *cry1* gene in response to host‐derived reactive nitrogen species (RNS) during infection via the ChrR‐RpoE pathway. This activation enhances bacterial resilience to environmental stressors, particularly blue light, by driving Cry1‐mediated DNA repair mechanisms.

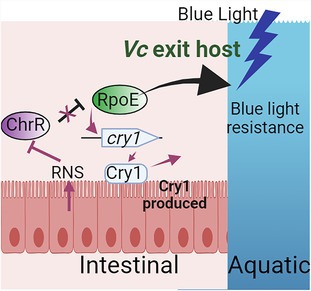

## 1 Introduction

*Vibrio cholerae*, a Gram‐negative bacterium, is the causative agent of cholera, leading to acute watery diarrhea (Clemens et al. 2017). Outside of the host, *V. cholerae* resides in estuarine and coastal environments, and it has adopted multiple strategies to combat environmental challenges (Colwell 1996). For example, *V. cholerae* has evolved robust response mechanisms to mitigate the potential adverse impacts of light. While photosynthetic organisms harness light for energy conversion, light can also cause photo‐oxidative damage to living cells, negatively affecting nucleic acids, lipids, and proteins (Elias‐Arnanz, Padmanabhan, and Murillo 2011). Given that UV radiation (100–380 nm wavelength) and blue light (380–470 nm) can effectively penetrate aquatic ecosystems (Häder et al. 1998), *V. cholerae* , much like many of its marine counterparts, has developed mechanisms for sensing these wavelengths of light (Braatsch and Klug 2004). This adaptation triggers the activation of a set of genes that encode proteins involved in photo‐oxidative responses. Three such proteins have been characterized in the *V. cholerae* genome that belong to the cryptochrome/photolyase family (CPF): VCA0057 (Phr), a cyclobutane pyrimidine dimer (CPD) photolyase; VC1814 (Cry1), and VC1392 (Cry2), the latter two being ssDNA‐specific photolyases (Selby and Sancar 2006). Transcriptomic analysis revealed the profound impact of blue light exposure on *V. cholerae* as 6.3% of the microbe's genes are differentially expressed in response to this stimulus (Tardu, Bulut, and Kavakli 2017). These three photolyase genes are among the highly induced genes.

The established transmission model for *V. cholerae* involves ingestion of contaminated water sources, followed by infection of the host, culminating in the return to aquatic reservoirs (Nelson et al. 2009; Hsiao and Zhu 2020). To establish colonization and cause disease, *V. cholerae* employs intricate signal transduction pathways to activate virulence factors (Matson, Withey, and DiRita 2007; Hsiao and Zhu 2020). Alongside activating virulence genes, *V. cholerae* must also express factors necessary to survive toxic compounds produced by the host, including nitric oxide (NO). Levels of NO, a free radical, surge during infection, disrupting proteins containing cysteine residues, iron‐dependent enzymatic reactions, and components of the electron transport chain (Poole 2005). In the host, NO is generated through acidified nitrite in the stomach and by enzymes like inducible nitric oxide synthase (iNOS) during inflammatory responses (Fang and Vazquez‐Torres 2019). Cholera patients exhibit heightened iNOS expression in their small intestines (Janoff et al. 1997; Qadri et al. 2002; Rabbani et al. 2001; Chen et al. 2022), implying that *V. cholerae* encounters NO during human infection. To counteract the detrimental effects of reactive nitrogen species (RNS), *V. cholerae* employs a transcriptional regulator NorR to sense NO and activate *hmpA*, encoding a member of the flavohemoglobin family of enzymes that catalyzes the conversion of NO to nitrous oxide or nitrate (Stern et al. 2012). NorR also activates the expression of *nnrS*, which encodes a novel protein that is important for RNS resistance (Stern et al. 2012). Additionally, NO impacts cellular signaling through S‐nitrosylation of protein cysteine residues, including the key virulence regulator AphB (Chen et al. 2022).

*V. cholerae* has evolved adaptive mechanisms to thrive across various challenging conditions during its transition from aquatic environments to the host gut and beyond. For instance, during initial infection, *V. cholerae* senses host signals and orchestrates both activation of virulence genes and repression of a set of genes that contributes to host defense evasion (Hsiao et al. 2006; Liu et al. 2008; Yang et al. 2013; Cakar et al. 2018). Late in infection, *V. cholerae* activates a set of “late induced genes” to facilitate its dissemination into the aquatic environment. However, the subsequent role of specific genes expressed during human infection in facilitating *V. cholerae* survival in its natural environment is less well known. In this study, we demonstrated that the photolyase Cry1 was critical for *V. cholerae* survival under blue light exposure. The expression of *cry1* was induced by blue light but also upregulated in the presence of NO. We showed that pre‐induction of Cry1 by NO prior to exposure to blue light confers a survival advantage upon *V. cholerae* possessing functional Cry1. We speculate that this NO‐induced upregulation of Cry1 plays an important role in *V. cholerae* environmental survival: *V. cholerae* encounters host‐derived NO, and the subsequent induction of Cry1 effectively primes the bacterium for the aquatic environmental challenges, where blue light is abundant, once it exits the host gut.

## 2 Results

### 2.1 Photolyase Cry1 Is Critical for *V. cholerae* Survival Under Blue Light Exposure

**FIGURE 1:**
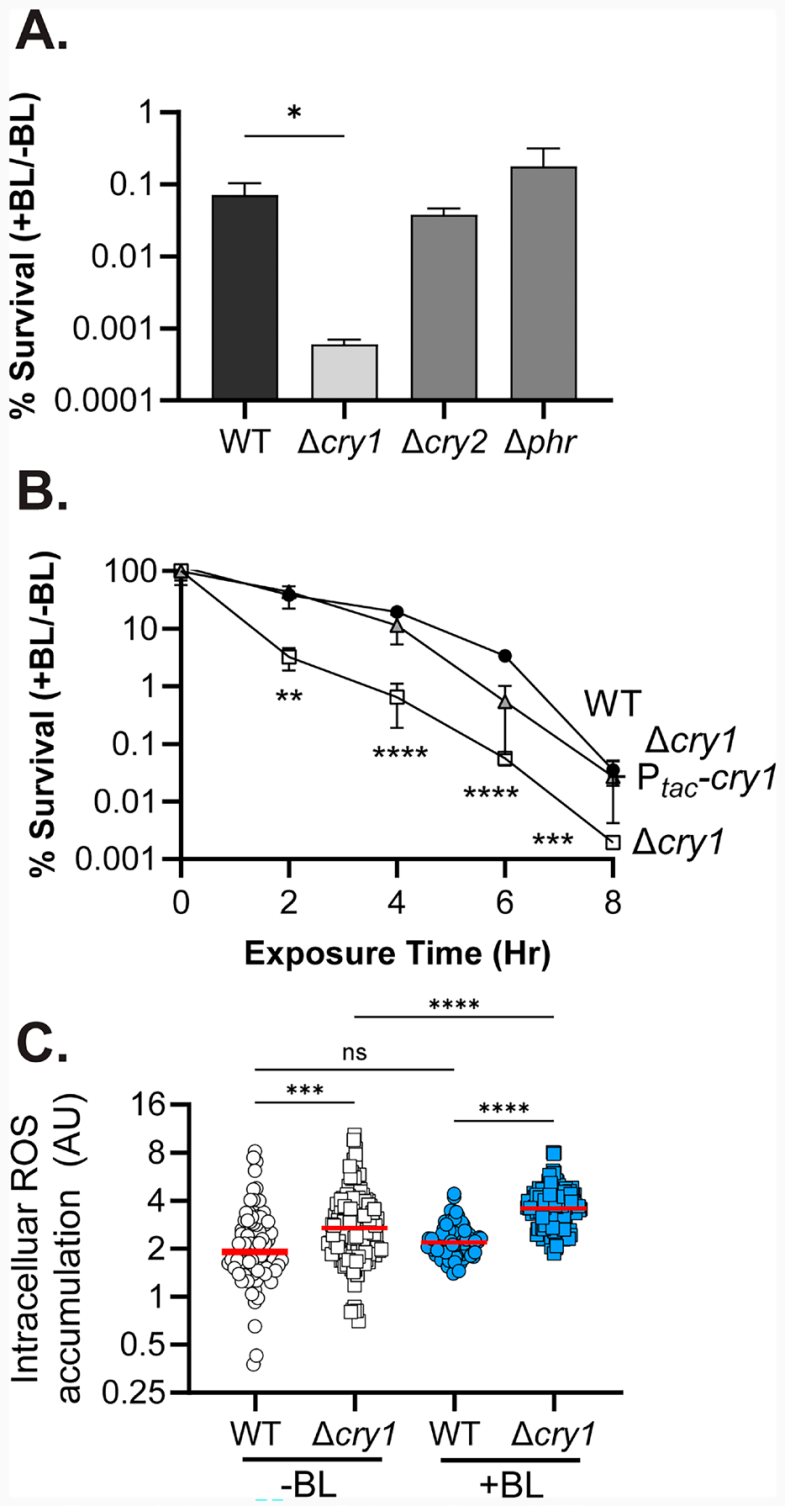
The effect of Cry1 on *V. cholerae* viability under blue light exposure. (A) Photolyase Tn mutants. Overnight cultures of wildtype and transposon insertion mutants of *cry1*, *cry2*, and *phr* were inoculated in artificial seawater at approximately 10^6^ cfu/mL. The samples were either exposed to blue light (+BL) or shielded with white tape (−BL control) for 8 h. Viable cells were determined by serial dilutions and plating. The data represent the mean and standard deviation from three independent experiments. *p < 0.05 (unpaired *t*‐test). (B) *cry1* in‐frame deletion, wildtype (with vector), Δ*cry1*(vector) and Δ*cry1*(P_*tac*_‐*cry1*) were inoculated in artificial seawater and exposed to blue light or shielded. At the time points indicated, viable cells were determined. The data shown represent means ± SD from four repeats. **p < 0.005; ***p < 0.0005; ****p < 0.0001 (determined by *t*‐test and compared to wildtype at each time point). (C) Intracellular ROS accumulation. Mid‐log‐phase cultures of wildtype or Δ*cry1* mutants containing chromosomal P_*tet*_‐*mCherry* reporters were resuspended in PBS buffer and either covered (−BL) or exposed to blue light (+BL) for 2 h followed by treatment with 10 μM DCFDA for 30 min. Fluorescent signals and mCherry intensity of each *V. cholerae* cell were measured by microscopy. Approximately 60–100 cells were analyzed for each condition, and the red lines indicate the mean of fluorescent intensity normalized against mCherry intensity of individual cells. ***p < 0.0005, ****p < 0.0001 (determined by one‐way ANOVA).

Since photolyases are known to repair cellular DNA damage caused by UV and blue light exposure (Worthington et al. 2003), we used blue light to investigate how these enzymes contribute to *V. cholerae* survival under such conditions. We constructed an apparatus delivering uniform blue light with a wavelength of 465 nm and an irradiance of approximately 200 mW/cm^2^, an intensity that is within the range *V. cholerae* may encounter in aquatic environments (Grant and Slusser 2004; Yamashita and Yoshimura, 2019). We diluted *V. cholerae* wildtype and transposon insertional mutants (Cameron et al. 2008) of *cry1*, *cry2*, and *phr* into artificial seawater (Joelsson et al. 2007) and exposed them to blue light for 8 h. Compared to the wildtype, the *cry1* mutant exhibited increased susceptibility to blue light, whereas the *cry2* and *phr* mutants did not (Figure 1A). These results suggest that Cry1 plays a crucial role in *V. cholerae* survival under blue light exposure. Of note, the expression of all three photolyase genes has been reported to be induced by blue light exposure (Tardu et al. 2017). However, it is possible that due to the differences in substrate recognition and DNA repair efficiency, only Cry1 is important under the tested condition.

We then constructed an in‐frame deletion in *cry1*. We found that compared to wildtype, survivability of viable Δ*cry1* cells significantly decreased with increased duration of blue light exposure (Figure 1B). When *cry1* was introduced on a plasmid in the Δ*cry1* mutant, the complemented strain exhibited comparable survivability to wildtype when exposed to blue light (Figure 1B). These data confirmed the important role of Cry1 in protecting *V. cholerae* from blue light. Since blue light exposure induces photo‐oxidative stress in bacteria (Elias‐Arnanz et al. 2011), we then examined the effect of Cry1 on blue light‐induced reactive oxygen species (ROS) accumulation in *V. cholerae* . Mid‐log phase cultures of wildtype or Δ*cry1* mutants were resuspended in PBS buffer containing 10 μM of a redox‐sensitive, cell‐permeable dye, 2′,7′‐dichlorodihydrofluorescein diacetate (DCFDA). The cells were either covered or exposed to blue light for 2 h, and fluorescence was examined by microscopy. Compared to wildtype, more ROS was accumulated in Δ*cry1* cells regardless of blue light exposure (Figure 1C). Blue light exposure further increased ROS accumulation in Δ*cry1* cells (Figure 1C). It has been reported that a mutation in the photolyase ortholog, PhrB, in *Neisseria gonorrhoeae* leads to increased sensitivity to oxidative stress (Cahoon et al. 2011). It is possible that Cry1 may play a similar role in resistance to ROS.

**FIGURE 2:**
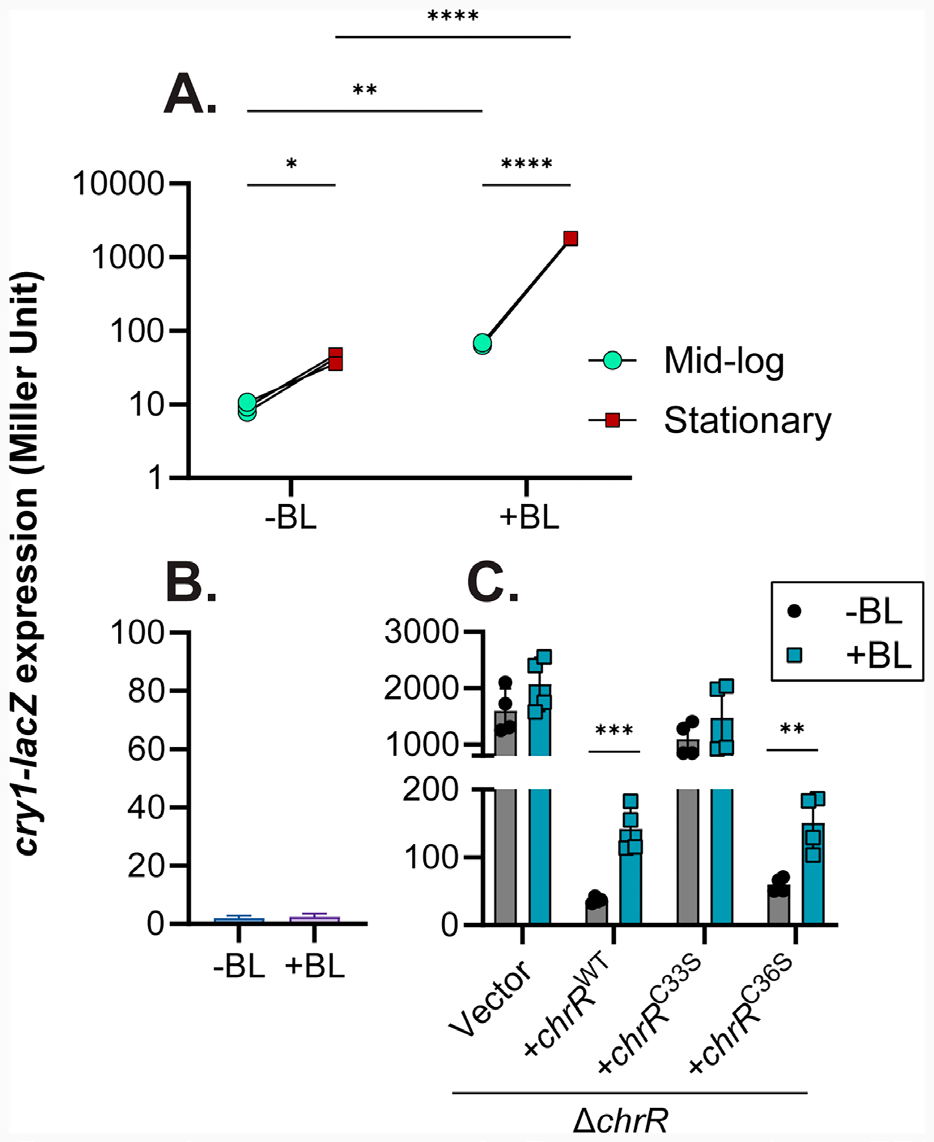
Blue light and NO induction of *cry1*. (A) Activation of *cry1* by blue light. Overnight cultures of wildtype containing a P*cry1‐lacZ* plasmid were inoculated into modified M9 medium. The cultures were either shielded (−BL control) or exposed to blue light (+BL). Cells were collected at mid‐log phase (6 h) and stationary phase (24 h) for β‐galactosidase activity assays. The data shown are means ± SD from four independent experiments. *p < 0.05, **p < 0.0005; ****p < 0.0001 (determined by unpaired *t*‐test). (B, C) The roles of RpoE and ChrR in *cry1* expression. Overnight cultures of Δ*rpoE* (B) or Δ*chrR* (vector), Δ*chrR (*P_*tac*_ *‐chrR* ^WT^ and cysteine→serine derivatives). (C) Cells harboring a *cry1‐lacZ* reporter plasmid were inoculated in modified M9 medium supplemented with 1 mM IPTG. The cultures were exposed to blue light for 6 h. The data shown represent means ± SD from four repeats. **p < 0.005; ***p < 0.0005 (determined by unpaired *t*‐test).

### 2.2 ChrR‐Mediated Blue Light Induction of *cry1*

To investigate how *cry1* expression is regulated, we constructed a *cry1‐lacZ* transcriptional reporter plasmid and introduced it into wild‐type *V. cholerae* . Overnight cultures of the resulting strain were diluted into M9 minimal medium and subsequently exposed to blue light. Samples were collected from mid‐log and stationary‐phase cultures, and β‐galactosidase activity was measured. In the absence of blue light, *cry1* expression remained low (Figure 2A). However, upon blue light exposure, *cry1* expression was induced, reaching its highest levels during stationary phase (Figure 2A).

A previous RNA‐seq study revealed that sigma factor E (RpoE) and anti‐sigma factor ChrR play a pivotal role in orchestrating transcriptional induction for over 150 genes in response to blue light, including *cry1* in *V. cholerae* (Tardu et al. 2017). To examine the direct involvement of RpoE and ChrR in *cry1* expression, we measured β‐galactosidase activity of *cry1‐lacZ* in Δ*rpoE* and Δ*chrR* mutants with or without blue light exposure. We observed a complete loss of induction of *cry1* in the Δ*rpoE* mutant (Figure 2B), and constitutive expression of the same in the Δ*chrR* mutant (Figure 2C). These data suggest that the ChrR‐RpoE regulatory pathway govern the expression of *cry1*.

*V. cholerae* ChrR is an ortholog of ChrR from the photosynthetic bacterium *Rhodobacter sphaeroides* . In *R. sphaeroides* , ChrR functions as a sensor for singlet oxygen stress arising from photosynthesis, leading to the release of its associated sigma factor, RpoE. This cascade results in the activation of the RpoE regulon (Anthony et al. 2005; Ziegelhoffer and Donohue, 2009). Both *V. cholerae* ChrR and *R. sphaeroides* ChrR belong to the family of zinc‐binding anti‐sigma factor (ZAS) proteins, featuring a conserved His_XXX_Cys_XX_Cys sequence motif. In *R. sphaeroides* , these two cysteine residues play a critical role in zinc binding, singlet oxygen sensing, and anti‐sigma E activity. To investigate the role of the cysteine residues in *V. cholerae* ChrR, we introduced site‐directed mutations to replace the coding sequences of ChrR C33 and C36 with serine residues. These mutated constructs were then cloned into a P_*tac*_‐controlled plasmid and introduced into Δ*chrR* mutants to assess their functionality by measuring *cry1‐lacZ* expression. We found that complementation with wildtype ChrR (ChrR^WT^) led to a reduction in the basal level expression of *cry1‐lacZ*, with subsequent induction by blue light (Figure 2C). In the strain expressing ChrR^C33S^, *cry1* expression was elevated regardless of blue light exposure, while in the ChrR^C36S^‐expressing strain, the blue light‐induced *cry1* expression pattern resembled that of the wildtype (Figure 2C). These findings suggest that in *V. cholerae* , the C33 residue plays a pivotal role in mediating ChrR's anti‐sigma E activity, as well as sensing blue light‐induced stress.

### 2.3 Nitric Oxide Induces *cry1*

**FIGURE 3:**
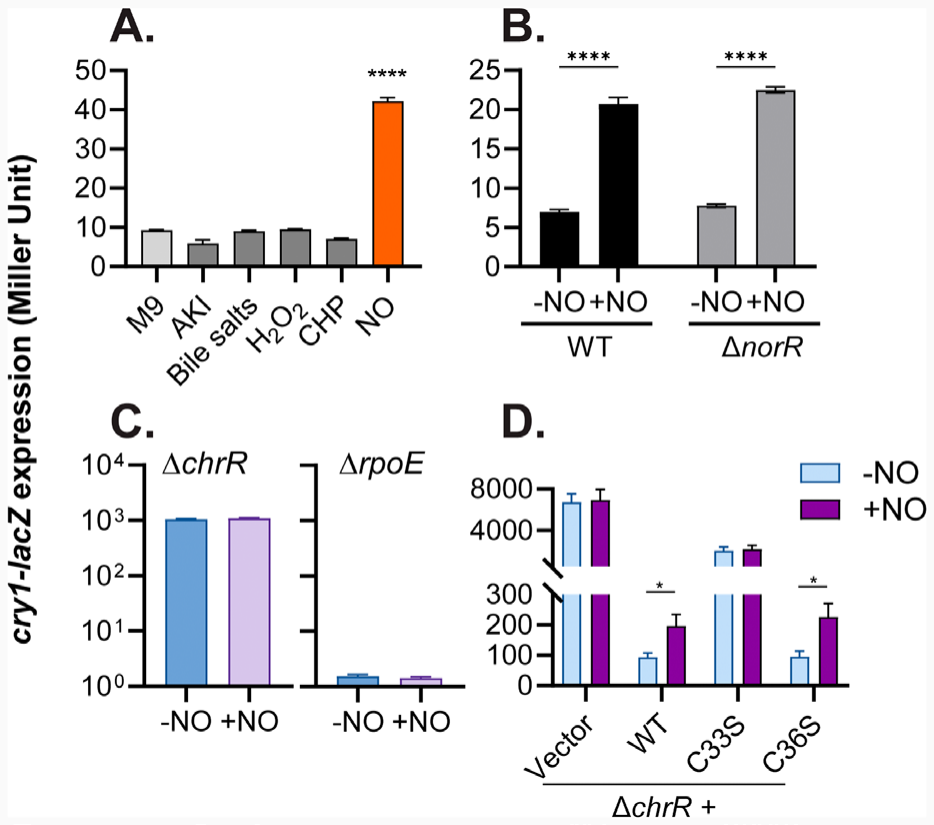
Regulation of NO‐mediated *cry1* induction. (A) Nitric oxide induces *cry1*. Overnight cultures of wildtype *V. cholerae* harboring a *cry1‐lacZ* plasmid were inoculated into modified M9 medium or AKI or minimal medium supplemented with hydrogen peroxide (100 μM), cumene hydroperoxide (25 μM), taurocholate (0.1%) or DEA NONOate (100 μM) and incubated statically at 37°C for 12 h. The data represent the mean and standard deviation from three independent experiments. ****p < 0.0001(unpaired *t*‐test). (B–D). Wildtype and mutants harboring a P*cry1‐lacZ* plasmid were inoculated into modified M9 medium at approximately 10^6^ cfu/mL with appropriate antibiotics with or without 100 μM DEA NONOate and incubated stationarily at 37°C for 12 h. For strains with plasmids bearing wild‐type *chrR* or its cysteine‐to‐serine mutants under the control of P_tac_, 1 mM IPTG was added to the medium (D). After incubation, cells were collected for β‐galactosidase activity assays. The data represent the mean and standard deviation from three independent experiments. *p < 0.05; ****p < 0.0001(unpaired *t*‐test).

A previous RNA‐seq study (Mandlik et al. 2011) reported that *cry1* expression is induced 16‐fold in *V. cholerae* colonizing mice compared to growth in LB, suggesting that *cry1* is expressed during infection even in the absence of light exposure. To identify the signals that activate *cry1* expression in vivo, we exposed *V. cholerae* containing the *cry1‐lacZ* reporter plasmid to various conditions that *V. cholerae* may encounter during infection. As shown in Figure 3A, *cry1* was not induced in AKI medium, a condition known to induce virulence gene expression in *V. cholerae* El Tor strains (Iwanaga and Yamamoto, 1985). Similarly, no induction was observed in the presence of bile salts, which activate the virulence regulator TcpP (Yang et al. 2013), nor with H₂O₂ or cumene hydroperoxide, both of which lead to producing reactive oxygen species that *V. cholerae* may encounter during infection (Wang et al. 2018; Liu et al. 2016). However, *cry1* was induced by nitric oxide (NO) (Figure 3A), an RNS produced by the host (Janoff et al. 1997; Qadri et al. 2002; Rabbani et al. 2001; Chen et al. 2022).

Next, we investigated the mechanism by which NO induces *cry1* expression. Previous studies have elucidated the key role of the transcriptional regulator NorR in sensing NO and activating genes involved in NO detoxification (Stern et al. 2012, 2013). To determine whether NorR is also essential for *cry1* transcriptional activation, we evaluated *cry1‐lacZ* expression in Δ*norR* mutants exposed to NO. We observed that NO‐induced *cry1* expression in Δ*norR* mutants was comparable to that in the wildtype strain (Figure 3B). These results suggest that the activation of *cry1* by NO occurs independently of NorR.

To determine whether RpoE and ChrR are involved in NO‐induced *cry1* expression, we measured *cry1‐lacZ* in Δ*rpoE* and Δ*chrR* mutants in the presence of NO. We found that *cry1* expression was significantly reduced in the Δ*rpoE* mutant and was constitutively elevated in the Δ*chrR* mutant (Figure 3C). Additionally, in the Δ*chrR* strain expressing ChrR^C33S^, *cry1* expression remained elevated irrespective of NO presence. In contrast, the Δ*chrR* strain expressing ChrR^C36S^ exhibited NO‐induced *cry1* expression similar to the wildtype (Figure 3D), suggesting that similar to sensing blue light, ChrR's C33 residue is essential for sensing NO and regulating *cry1* expression.

### 2.4 Cry1 Pre‐Induced by NO In Vivo Is Important for *V. cholerae* Blue Light Protection

**FIGURE 4:**
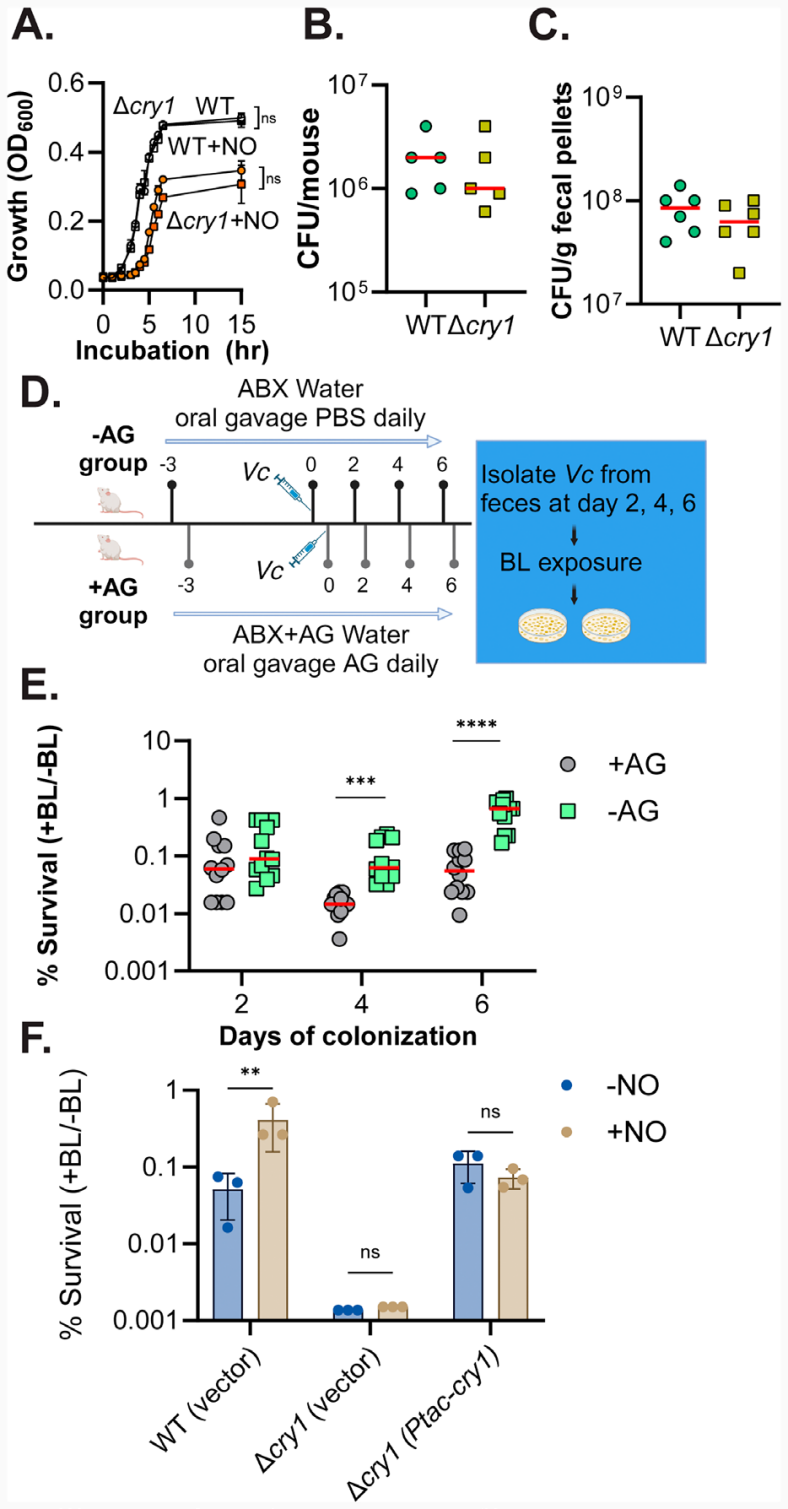
Influence of Cry1 pre‐induction on *V. cholerae* survival under blue light exposure. (A) NO resistance. Wildtype (circles) and Δ*cry1* mutant (squares) were grown in modified M9 medium without (open) or with (orange) 500 μM DEA NONOate. OD_600_ was recorded. The data shown represent means ± SD from four repeats. (B, C) Colonization of wildtype and Δ*cry1* in the infant mouse model (B). and the adult mouse model (C). (D) Schematic diagram of AG‐treated mouse experiments. ABX water includes 1 mg/mL streptomycin and 0.5% aspartame. AG: Aminoguanidine, administered at 1 mg/mL in water with daily 1 mg/mouse intragastrical inoculation. (E) *V. cholerae* blue light sensitivity after exiting from mice. Fecal pellets were collected and purified 2, 4, and 6 days after *V. cholerae* mouse inoculation. Approximately 10^6^ CFU/mL *V. cholerae* cells were then diluted into artificial seawater. The cultures were either covered or exposed to blue light for 6 h, and viable cells were quantified. ***p < 0.005; ****p < 0.0001 (determined by unpaired *t*‐test). (F) In vitro *V. cholerae* cells were incubated in minimal media with or without 100 μM of DEA NONOate for 16 h and then diluted into artificial seawater, shielded or exposed to blue light for 8 h. Percentage survival was determined by normalizing the exposed cells to non‐exposed cells. The data shown represent means ± SD from three repeats. **p < 0.0105 (determined by unpaired *t*‐test).

To explore the potential involvement of Cry1 in the pathogenesis of *V. cholerae* , we first tested whether Cry1 contributes to NO resistance in *V. cholerae*. We constructed an in‐frame deletion of *cry1*. When we exposed *V. cholerae* to high concentrations of NO donors (up to 500 μM), we observed minimal growth differences between the wildtype and Δ*cry1* mutants (Figure 4A). These data suggest that Cry1 is not critical for *V. cholerae* RNS resistance.

Next, we assessed the colonization capabilities of Δ*cry1* mutants in comparison to the wildtype strain. Our findings revealed that the colonization efficiency of Δ*cry1* mutants was comparable to that of the wildtype in both the infant mouse model (Figure 4B) and the streptomycin‐treated adult mouse model (Figure 4C). These results suggest that the Cry1 protein is not essential for *V. cholerae* to colonize the host intestines.

During infection, *V. cholerae* is known to encounter host‐derived NO (Janoff et al. 1997; Qadri et al. 2002; Rabbani et al. 2001; Chen et al. 2022). Given that the *cry1* gene is induced in the presence of NO (Figure 3), we proposed that NO‐mediated *cry1* induction might shield *V. cholerae* from blue light damage after it exits the host and enters aquatic environments. To test this hypothesis, we used a streptomycin‐treated adult mouse model, in which bacteria experience host‐generated oxidative and nitrosative stress. Mice were also treated with PBS control or aminoguanidine (AG), an iNOS inhibitor that reduces NO production in mice (Cross et al. 1994; Winter et al. 2013) (Figure 4D). Two, four, and six days post‐infection, fecal samples were collected from each group. *V. cholerae* was purified by differential centrifugation, and then introduced into seawater environments, with some samples exposed to blue light. The results showed a significantly higher survival rate of *V. cholerae* exposed to RNS (from the −AG mice group) under blue light compared to those from the RNS‐mitigated (+AG) group (Figure 4E). This indicates that host‐derived RNS during infection stimulates a protective response against the harmful effects of blue light after leaving the host.

To validate this phenomenon in vitro, we simulated conditions of NO preinduction. *V. cholerae* was cultured overnight in LB medium, with and without NO supplementation. The bacteria were then diluted in artificial seawater and exposed to blue light for 8 h. This experiment revealed that NO pre‐incubation significantly increased the blue light survival rate of *V. cholerae* by approximately tenfold (Figure 4F), supporting the hypothesis that *cry1* pre‐induction enhances survival against blue light in aquatic environments.

## 3 Discussion

In this study, we found that Cry1, a DNA repair photolyase, is critical for *V. cholerae* to resist blue‐light‐induced stress, a challenge frequently encountered in its aquatic environment. The regulation of *cry1* by RpoE and its anti‐sigma factor ChrR adds an additional layer of complexity to *V. cholerae* 's stress response network. A previous RNA‐seq study (Tardu et al. 2017) demonstrated that genes such as glutaredoxin (VC2044) and glutathione S‐transferase (VC1096) that are involved in resistance to reactive oxygen species (ROS), a common environmental stimulus in aquatic habitats, are also induced by the ChrR‐RpoE system; suggesting broader roles of the RpoE regulon in the aquatic environment (Tardu et al. 2017). Although *V. cholerae* is insulated from light during infection, our data reveal that *cry1* expression is activated not only by blue light, but also by nitric oxide (NO), a host‐derived reactive nitrogen species. Intriguingly, we identified one of the two cysteine residues (C33) in ChrR as critical for sensing RNS and modulating *cry1* expression. This dual regulation exemplifies how bacteria integrate host‐derived and environmental signals to adapt and thrive in diverse conditions.

**FIGURE 5:**
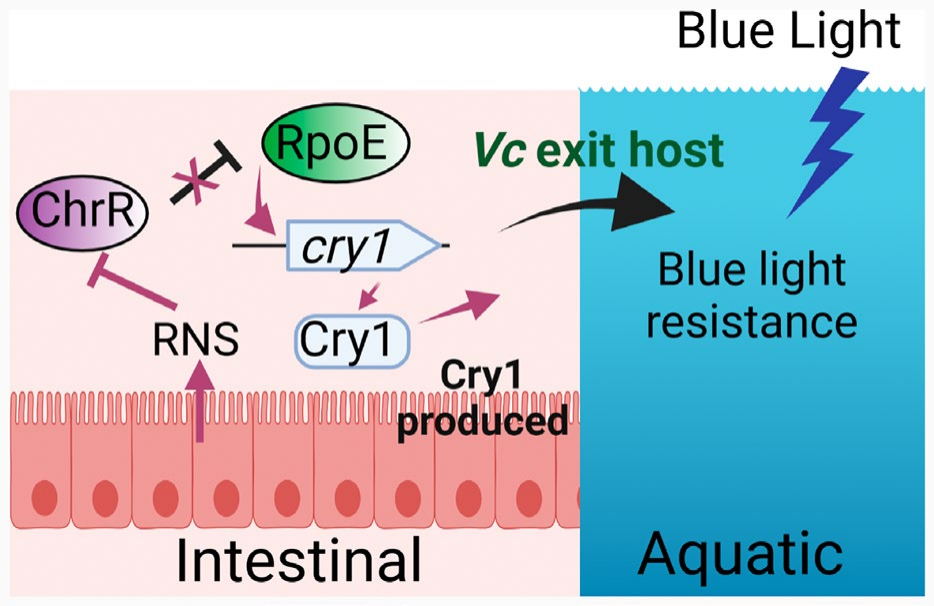
Enhanced environmental survival of *V. cholerae* through in vivo‐induced Cry1: A working model. During *V. cholerae* infection, the activation of *cry1* by host‐derived RNS occurs via the ChrR‐RpoE pathway. This activation primes *V. cholerae* to confront aquatic environmental challenges, including blue light exposure, which sustains Cry1 expression.

We propose a working model (Figure 5) where *V. cholerae* alternates between host colonization and survival in aquatic ecosystems. During late‐stage infection, host‐derived RNS, including NO, modify the anti‐sigma factor ChrR, leading to RpoE activation and upregulation of *cry1*. This priming event equips the bacterium to resist blue light stress in aquatic environments. Once in the environment, continued exposure to blue light sustains *cry1* expression via the same ChrR‐RpoE regulatory pathway. This mechanism illustrates how host signals can prepare bacteria for environmental stressors, providing a significant fitness advantage during transitions.

Light is a fundamental environmental factor, and its role in bacterial physiology extends beyond phototrophic systems. Even some chemotrophic bacteria encode photoreceptor proteins, enabling them to detect and respond to changes in light environments (Purcell et al. 2007). These responses regulate critical lifestyle decisions, such as transitions between motile, single‐cellular states and surface‐attached biofilms, or between environmental and host‐associated niches (Elias‐Arnanz et al. 2011; Gomelsky and Hoff 2011). Similarly, *V. cholerae* 's use of Cry1 to mitigate blue light stress—regulated by both host and environmental cues—aligns with this trend and underscores the versatility of bacterial photoregulation. Further studies are needed to understand the specific role of Cry1 in natural aquatic ecosystems, where additional stressors such as oxidative stress coexist. Additionally, whether Cry1 integrates with other known light‐sensing systems or biofilm‐related pathways in *V. cholerae* remains unknown. Exploring the interplay between Cry1 and other regulatory pathways, such as cyclic di‐GMP or quorum sensing, could reveal broader implications for bacterial persistence and community behavior.

### 3.1 Limitations of the Study

We recognize several limitations in our study. First, we used a commercially available kit with DCFH‐DA dyes to measure ROS accumulation in wildtype and *cry1* mutants under blue light exposure or in its absence. This method, while widely used, may not provide fully accurate measurements as it broadly detects oxidizing species, lacks selectivity, and is prone to experimental artifacts (Imlay, 2015; Murphy et al. 2022). Additionally, we employed DEA NONOate, a nitric oxide donor with a half‐life of approximately 2 min, as our NO source. As a result, the NO concentrations reported throughout the paper may not precisely represent the actual NO levels during the experiments. Finally, further studies are required to elucidate the precise role of C33 in ChrR that regulates this activity.

## 4 Methods and Materials

### 4.1 Strains and Culture Conditions

*V. cholerae* El Tor C6706 (Zhu et al. 2002) was used as the wildtype strain throughout this study. Both *V. cholerae* and *E. coli* were propagated in LB (Luria‐Bertani) Miller medium with appropriate antibiotics at 37°C, unless otherwise noted. The minimal medium used in this study was a modified M9 medium. It contained the standard M9 medium components supplemented with 0.2% glucose, 10 mM HEPES (pH 7.4), and 0.1% casamino acids. Artificial seawater used in this study was described previously (Joelsson et al. 2007). Diethylammonium (Z)‐1‐(N,N‐diethylamino)diazen‐1‐ium‐1,2‐diolate (DEA NONOate) (Cayman Chemical) was used as the NO donor. When overnight cultures were inoculated into a new medium, 1000‐fold dilution was used.

The strains and primers used in this study are listed in Table 1. In‐frame deletions of *cry1* (VC1814), *chrR* (VC2301), and *rpoE* (VC2302) were generated using the multiplex genome editing by natural transformation (MuGENT) method (Dalia et al. 2014). Complementation of *cry1* was constructed by PCR amplification of *cry1* coding sequences, followed by cloning it into the pSRKTc vector, which harbors *lacI* ^Q^ and a P_*tac*_ promoter (Khan et al. 2008). Similarly, P_*tac*_‐controlled *chrR* wildtype and its cysteine‐to‐serine derivatives were constructed by cloning the respective sequences into pSRKTc. The *cry1‐lacZ* transcriptional reporter was constructed by cloning the *cry1* promoter region into pAH6 (Zhou et al. 2021). *V. cholerae* containing a constitutive P_tet_‐*mCherry* was constructed via homologous recombination of P_tet_‐*mCherry* into the intergenic region between VCA0104‐VCA0105 (Zhou et al. 2021). The *norR* deletion mutant was constructed as previously described (Stern et al. 2012).

**TABLE 1:** Strains, plasmids, and primers used in this study.

Strains	Characteristics	Source
*V. cholerae* O1 El Tor C6706	Wild type	Zhu et al. (2002)
C6706::P_*tet*_‐*mCherry*	P_tet_‐*mCherry* into the VCA0104‐VCA0105 intergenic region	Chen et al. 2022)
Δ*cry1*	In‐frame deletion of VC1814, VC1807::Sp^R^	This work
Δ*norR*	In‐frame deletion of VCA0182, Δ*lacZ*	Stern et al. (2012)
Δ*rpoE*	In‐frame deletion of VC2032, Δ*lacZ*, VC1807::Sp^R^	This work
Δ*chrR*	In‐frame deletion of VC2031, Δ*lacZ*, VC1807::Sp^R^	This work

Plasmids	Characteristics	Source
pSRKTc	lacI^Q^, P_*tac*_ promoter	Khan et al. (2008)
pAH6	Promoterless *lacZ* reporter plasmid	Zhou et al. (2021)
P*cry1*‐*lacZ*	pAH6::*cry1* promoter region	This work
P_*tac*_ *‐cry1*	pSRTKc::*cry1*	This work
P_*tac*_‐*chrR* ^WT^	pSRKTc::*chrR*	This work
P_*tac*_‐*chrR* ^C33S^	pSRKTc::*chrR* ^C33S^	This work
P_*tac*_‐*chrR* ^C36S^	pSRKTc::*chrR* ^C36S^	This work

Primers	
In‐frame deletion	
*cry1*	TGATCGTTGATCATACTATTGATTCTGA ACTCAAGTTCCACTCTCAATACC GGTATTGAGAGTGGAACTTGAGTTCATGAATATCGTGATCATCGGT GGCAACTCCAAGTGGTTCG
*rpoE*	CTTCAAGCGTCTGCTCTG GTCGACGGATCCCCGGAATATAAACTCATCCTTAATGACTCAGG GAAGCAGCTCCAGCCTACAAGTTATCACCCTAATTATGCTTTAC GAAGCACTCAATCATGGAAAAC
*chrR*	TAAAAACGTCTGCGATAAAATCAC GTCGACGGATCCCCGGAATGGGTGATAACTCATAAGGACTC GAAGCAGCTCCAGCCTACACGCCTTACTGGATGATTTTATCC TTAAACCCACAAGCGAAGATC
Plasmid construction	
*cry1‐lacZ*	GCAAAGCTTCACCAGATCTGGCTTATTCATGGC GCAGTCGACCGTTCACTCGTAAATCGTTGGTAAAC
P*tac‐cry1*	ATGTCTAGACCGCCGAGTTGGTTATCAC ATGCATATGCTGATCCCCTTTAAAATCGGC
P*tac‐chrR*	GAATTCCATATGGAGTTATCACCCTAATTATGC GATAAGCTTTTACGGGTAAAGTCCCTTG

### 4.2 Blue Light Irradiation of Bacterial Cultures

The blue light setup was created using 11 strips cut from a 5 m single‐color COB blue LED strip, each measuring 12 cm in length. These strips were linked together to ensure even light distribution and attached to a glass sheet with adhesive backing. The apparatus covered an irradiance area of 12 cm × 18.5 cm, emitting around 200 mW/cm^2^ of 465 nm blue light. It was positioned 12 cm above white 96‐well plates, to regulate irradiance and minimize heat‐related interference. Continuous operation yielded a temperature of approximately 30°C. For non‐blue light control cultures, 3 layers of tape were applied to shield the wells. All materials for constructing the blue light apparatus were sourced from Super Bright LEDs.

### 4.3 Measurement of *cry1* Expression Under Different Conditions

Overnight cultures of strains containing the P_*cry1*_‐*lacZ* reporter plasmid were inoculated in either AKI, or modified M9 minimal medium supplemented with chloramphenicol (2 μg/mL) with or without hydrogen peroxide (100 μM), cumene hydroperoxide (25 μM), sodium taurocholate (0.01%) or the NO donor DEA NONOate (100 μM). When testing strains containing the pSRKTc vector and derivatives, an additional 1 μg/mL tetracycline and 1 mM IPTG were included. The cultures were then incubated at 37°C without shaking for 12 h. β‐galactosidase activity assays were then performed (Miller 1972) and the results were reported as Miller Units.

To measure the blue light effects on *cry1* induction, overnight cultures were inoculated into modified M9 minimal medium and transferred to a white 96‐well plate for blue light exposure. At different time points indicated, cultures were retrieved and subjected to β‐galactosidase activity assay.

### 4.4 Bacterial Survivability Assays Under Different Stressors

For measuring blue light survival, overnight cultures of different *V. cholerae* strains were diluted into artificial seawater and transferred to wells of a white 96‐well plate for blue light exposure (see above). At different times indicated, samples were retrieved and viable CFU was determined by serial 10‐fold dilutions and plating on LB plates with appropriate antibiotics.

To examine how NO pre‐induction affects *V. cholerae* survivability under subsequent blue light exposure, overnight cultures of *V. cholerae* cells were inoculated in modified minimal medium with or without 100 μM DEA NONOate for 16 h at 37°C without shaking. Subsequently, the cells were transferred to artificial seawater and exposed to blue light for 8 h. Viable CFU was then determined as above.

The NO sensitivity assay was conducted by inoculating the overnight cultures of wildtype and Δ*cry1* into modified M9 minimal media with or without 500 μM DEA NONOate. The cultures were then incubated without shaking at 37°C and sampled for optical density measurements (OD_600_, Bio‐tek).

### 4.5 Intracellular ROS Accumulation

Intracellular ROS levels of *V. cholerae* were measured by staining bacteria with the redox‐sensitive, cell‐permeable dye 2′,7′‐dichlorodihydrofluorescein diacetate (DCFDA). Mid‐log phase cultures of wildtype and Δ*cry1* containing a chromosomal P_*tet*_‐mCherry insertion were resuspended in PBS buffer and exposed to blue light for 2 h. Cells were then treated with 10 μM DCFDA (Abcam) for 30 min. Cells were subsequently washed in PBS to remove the excess dye. GFP and mCherry signals, representing ROS accumulation and cell viability respectively, of each *V. cholerae* cell were measured using a Nikon NiU fluorescence microscope.

### 4.6 *V. cholerae* Mouse Colonization

All animal experiments were performed in strict accordance with the animal protocols that were approved by the IACUC of the University of Pennsylvania.

For in vivo competition assay using the infant mouse model (Liu et al. 2015), approximately 10^5^ CFU of each of the two differentially labeled strains (wildtype was kanamycin‐resistant and Δ*cry1* was spectinomycin‐resistant) were mixed at a 1:1 ratio and inoculated intragastrically into 5‐day‐old CD‐1 infant mice. After 16 h, small intestines were collected and homogenized. The colonized numbers of mutant and wildtype were determined by plating on LB agar plates containing either 50 μg/mL kanamycin or 100 μg/mL spectinomycin.

The streptomycin‐treated adult mouse model (Chen et al. 2022) was used to examine the effect of *cry1* preinduction in vivo. Briefly, six‐week‐old CD‐1 mice were used, and 0.5% (wt/vol) streptomycin and 0.5% aspartame were added to the drinking water throughout the experiment. After 3 days of streptomycin treatment, 1 mg/mL aminoguanidine (AG, Acros Organics) was added to the drinking water of +AG group of mice throughout the remainder of the experiment. Additionally, the equivalent of 1 mg of AG was orally gavaged into each mouse in the +AG group daily (or PBS in the −AG group) throughout the remainder of the experiment. One day after starting the AG treatment, approximately 10^8^ CFU wildtype *V. cholerae* were intragastrically administered to each mouse. Fecal pellets were collected from each mouse at the indicated time points, resuspended in PBS buffer. Fecal debris was removed and bacterial cells were partially purified by a differential centrifugation method described previously with modifications (Emerson and Cabelli, 1982). Briefly, the homogenized samples were carefully loaded to the top of 10 mL 20% polyethylene glycol 8000 (PEG‐8000) in a 15‐mL conical tube. The tubes were then centrifuged at 6000 rpm for 10 min. The cleared top layer was collected, and the procedure was repeated. Bacterial cells were then inoculated into artificial seawater for blue light exposure experiments. Simultaneously, the samples were serially diluted, and then inoculated on plates containing appropriate antibiotics.

## Author Contributions

**Arkaprabha Banerjee:** investigation, writing – review and editing, conceptualization, writing – original draft, validation, methodology. **Hyuntae Byun:** investigation, methodology, writing – review and editing, conceptualization, writing – original draft. **Andrew J. Hrycko:** investigation, methodology, writing – review and editing. **Qinqin Pu:** investigation. **Mary R. Brockett:** investigation. **Nathaniel C. Esteves:** writing – review and editing, investigation. **Jennifer R. Miller:** investigation. **Qiushi Li:** investigation. **Amy T. Ma:** writing – review and editing, investigation. **Jun Zhu:** conceptualization, funding acquisition, writing – original draft, methodology, visualization, writing – review and editing, supervision.

## Conflicts of Interest

The authors declare no conflicts of interest.

## Data Availability

The data that support the findings of this study are available from the corresponding author upon reasonable request.
